# Supplementary figures and images for: Identification and characterization of a new soybean promoter induced by Phakopsora pachyrhizi, the causal agent of Asian soybean rust
Source: BMC Biotechnol. 2021 Mar 25;21:27. doi: 10.1186/s12896-021-00684-9 (PMC7995590; doi:10.1186/s12896-021-00684-9)

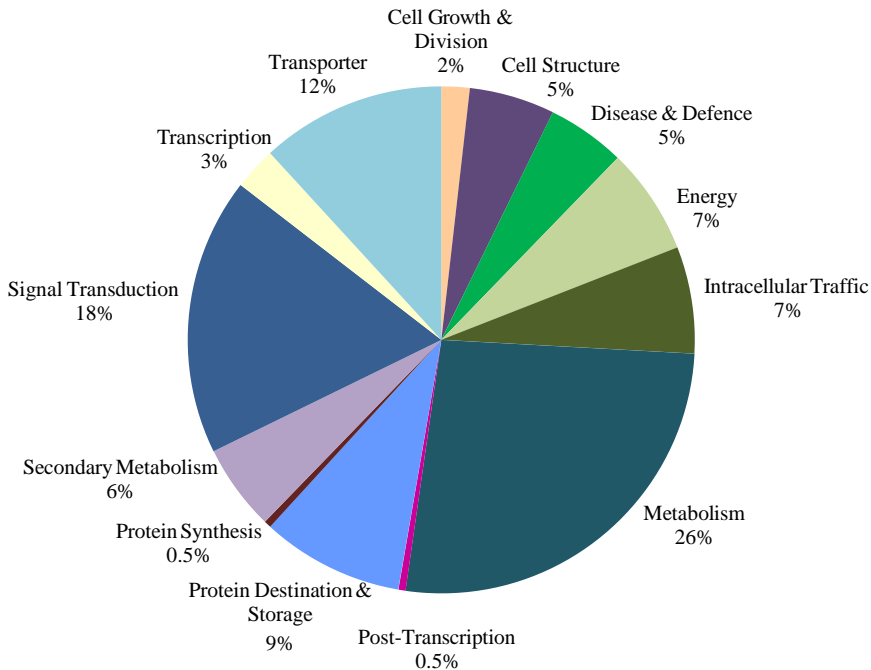

Supplement: Supplementary file 3 — Additional file 3: Figure S2. Relative expression of Glyma.13G346700 and Glyma.11G124500 compared to control plants (untreated) at 0.5, 1, 3, and 24 hours post chitinheptaose (DP7) treatment. Black boxes represent no change in genes expression compared with the control plants, red boxes indicate upregulation by DP7 treatment. The plants were sprayed with 200 ppm of DP7 or water until run-off. They were incubated in growth chamber temperature (24°C, 16 h light/8 h night, light intensity 15 μE.m-2.s-1 and 80% relative humidity) for 24 hours. [file 12896_2021_684_MOESM3_ESM.pdf]

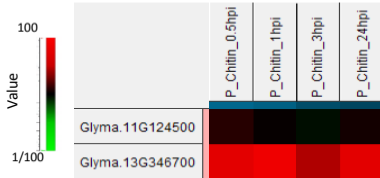

Supplement: Supplementary file 4 — Additional file 4: Figure S3. GmCHIT protein KRH29572.1 homologs, their functions and % of identity. From BLASTP analysis (NCBI). [file 12896_2021_684_MOESM4_ESM.pdf]

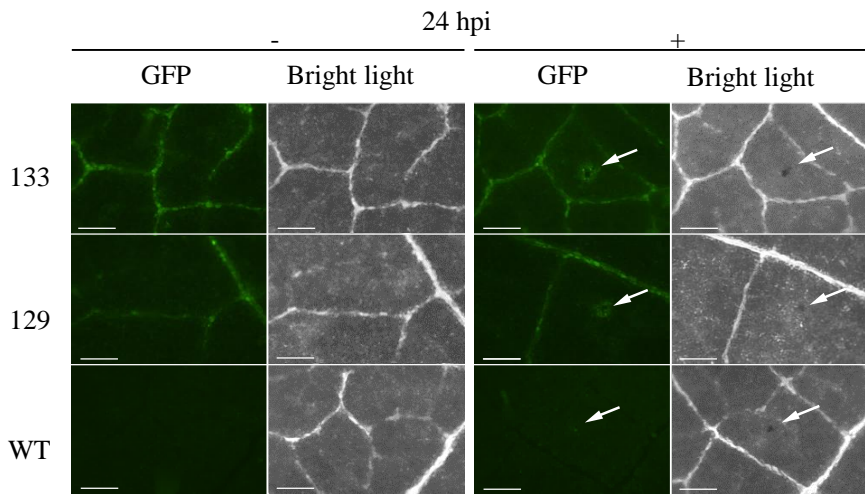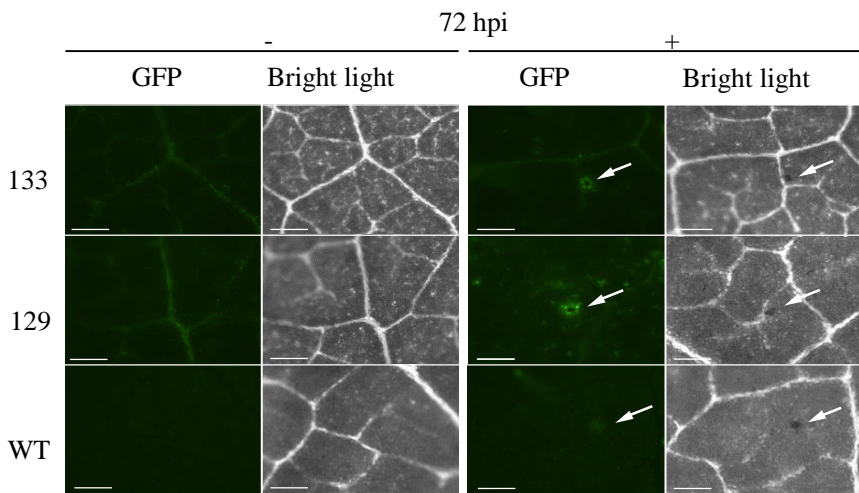

Supplement: Supplementary file 8 — Additional file 8: Figure S7. GmCHIT1 promoter expression following salicylic acid treatment. (a) GFP fluorescence in line 131 (pGmCHIT1:GFP) and WT detached leaves following SA (+) or mock (-) treatments. Graphics represent the fluorescence intensity measured with MetaMorph software via grayscale value. Mean of 20 biological replicates ± standard errors. No significant difference between treated (+) and untreated (-) leaves (Student’s t-test, p < 0.05). Representative images of the observed fluorescence are shown under the graphs. Bar-scales represent 5mm. Observations were realized at 24, 48 and 72 hours after hormonal treatment with a dissection scope (Leica Z16 APO) under GFP filter. (b) Relative expression of GmPR1 (GenBank: BU5773813), GmPR2 (GenBank: M37753), GmPR3 (GenBank: AF202731) in event 131 (pGmCHIT1:GFP) after SA treatment. Transcript accumulation at 24 and 48 hours compared to that in the mock-treated plants. The actin (GenBank: NM_001289231.2) and an elongation factor (GenBank: NM_001249608.2) encoding genes were used as references [32]. Three independent biological replicates ± standard deviations. *: significant difference between treated and untreated leaves determined by a Student’s t-test (p < 0.05). [file 12896_2021_684_MOESM8_ESM.pdf]

**a**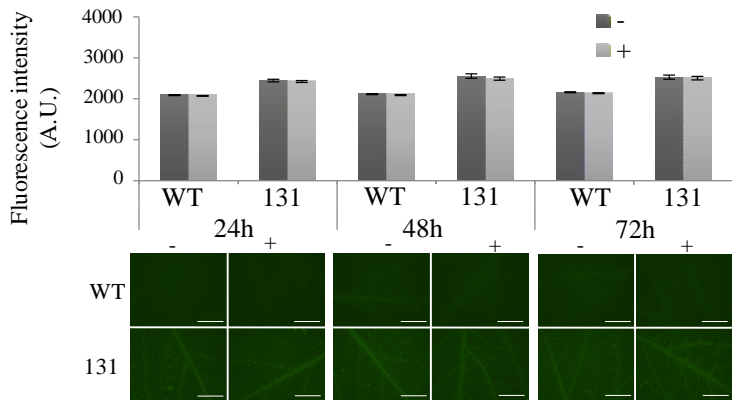**b**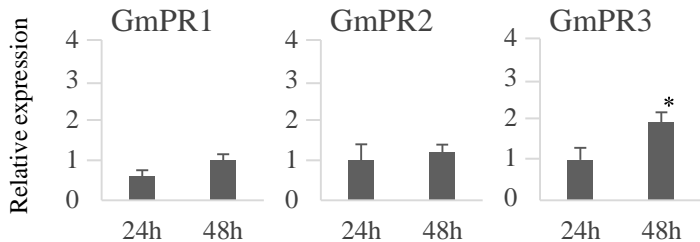

Supplement: Supplementary file 9 — Additional file 9: Table S2. Primers used for PCR and qPCR. *from Hirschburger et al., 2015 [32], ** from Mazarei et al., 2007 [64], *** from Zhong et al., 2014 [70]. [file 12896_2021_684_MOESM9_ESM.pdf]
