# Supplementary material for: Identification and characterization of a new soybean promoter induced by Phakopsora pachyrhizi, the causal agent of Asian soybean rust
Source: BMC Biotechnol. 2021 Mar 25;21:27. doi: 10.1186/s12896-021-00684-9 (PMC7995590; doi:10.1186/s12896-021-00684-9)
Supplement: Supplementary file 5 — Additional file 5: Figure S4. Multiple Sequence Alignment of GmCHIT (KRH29572.1) with plant homologous proteins (from BLASTP analysis). Similar residues are colored according to BLOSUM62 score: Max: 3.0 Low: 0.5. [file 12896_2021_684_MOESM5_ESM.pdf]

Proteins homolog	Predicted function	% of identity
Glycine soja KHN06178.1	**Endochitinase PR4**	100
Cicer arietium XP_012570981.1	**Chitinase**	85
Vigna angularis BAT90863.1	Hypothetical protein	82
Lotus japonicus AFK37127.1	Unknown	80
Phaseolus vulgaris XP_007131917.1	Hypothetical protein	80
Vigna radiata XP_022642874.1	Uncharacterized protein	78
Arachis hypogaea EP3 XP025686362	**Endochitinase**	75
Arachis ipaensis XP_016187096.1	**Endochitinase EP3**	75
Arachis duranensis XP015952096	**Endochitinase EP3**	75
Cajanus cajan XP_020224332.1	**Endochitinase PR4-like**	69
Medicago sativa ACL36992.1	**Chitinase class IV**	66
Medicago truncatula XP_003597548.2	**Endochitinase PR4**	66
Trifolium pratense PNY03482.1	**Endochitinase PR4-like**	64
