# Supplementary material for: Identification and characterization of a new soybean promoter induced by Phakopsora pachyrhizi, the causal agent of Asian soybean rust
Source: BMC Biotechnol. 2021 Mar 25;21:27. doi: 10.1186/s12896-021-00684-9 (PMC7995590; doi:10.1186/s12896-021-00684-9)
Supplement: Supplementary file 6 — Additional file 6: Figure S5. The GmCHIT1 promoter and potential cis-regulatory elements identified. (a) List of the cis-regulatory elements related to pathogen infection identified in the GmCHIT1 promoter. (b) Map of the GmCHIT1 promoter. AUX: auxin responsive element, GA: gibberellic acid responsive elements, MYB: MYB recognition elements, GT1-box: pathogen and NaCl responsive elements, W-box: pathogen responsive elements, TSS: transcription start site. [file 12896_2021_684_MOESM6_ESM.pdf]

1	XP_0071319	---------mskVAGFvaTlFMvvMMmMVPKteSAl------------------------
2	BAT90863.1	---MlqvamtskVAGFvtTlFMMvMM-MVPKtVSAe------------------------
3	XP_0226428	---MlqvamvskVAGFvtTlFMMvMMtMVPKteSs-------------------------
4	XP_0256863	-MrMekRLLl-------MgiwvMMg--MVk------snG---------------------
5	XP_0161870	MrmekrlLLkMcV----MgiwvMMg--MVk------------------------------
6	XP_0159520	-MrMekRLLl-------MgiwvMMg--MVk------snG---------------------
7	XP_0194143	MMnirNKLalI------ITFvvtiM--llsKdVSAQsCGCtEGLCCSQYGYCGtGDAYCG
8	PNY03482.1	MttMaNKsLnIsIAtlAISFFiMt---MVPKNVSAQNCGCAEGLCCSQYGYCGtsDdYCG
9	ACL36992.1	MtmMgNKsLsIcmAtlAIaFFiMi---MVPKNVSAQNCGCAEGvCCSQYGYCGNGDAYCG
10	XP_0035975	MtmMgNKsLsIcmAtlAIaFFiMi---MVPKNVSAQNCGCAEGvCCSQYGYCGNGDAYCG
11	AFK37127.1	-MsMgNKLLnIhaprFAVaFvMMM---MVPKfVrAQs-----------------------
12	XP_0125709	---MgNKLLkMc-----IaFFiMt---MVPKNViAQNCGCAEGLCCSQYGYCGNsDAYCG
13	XP_0202243	---MgyKLLcVlVA---VTFvMit---kVPKNVSAQNCGCAEGLCCSQYGYCGtGDAYCG
14	**KRH29572.1**	----msKLLlIrVAGFvaTFvtMvMM-MVPKNVSAQNiG---------------------
15	KHN06178.1	---------------------------MVPKNVSAQNiG---------------------
16	XP_0071319	------------------------hiADIVTrqFFNnIInkADgdCpGKsFYSRDAFLkA
17	BAT90863.1	------------------------NidrIVTQqFFdmIInQADgdCpGKNFYSRDAFLkA
18	XP_0226428	------------------------hidkIVTpqFFdmIInQADgdCpGKNFYSRDAFLkA
19	XP_0256863	------------------------sVgDIVTQeFFNGIIDeADdSCAGKkFYSRDgFLsA
20	XP_0161870	---------------------sDgsVgDIVTQeFFNGIIDeADdSCAGKkFYSRDAFLsA
21	XP_0159520	------------------------sVgDIVTQeFFNGIIDeADdSCAGKkFYSRDAFLsA
22	XP_0194143	TGCKeGPCYAspSeP------NDVNVADIVTpeFFNGIIDeADSSCeGKNFYSRDAFLNA
23	PNY03482.1	TGCKeGPCtSGtiPPSsPspsNDVNVADIVTQDFFNGIIDQADSSCAGKsFYtRaAFLdA
24	ACL36992.1	TGCKQGPCYAGQTPPSlPN--NDaNVADIlTQDFFNsIIDQADSSCAGKNFYtRaAFLdA
25	XP_0035975	TGCKQGPCYAGQTPPSlPN--NDaNVADIlTQDFFNrIIDQADSSCAGKNFYtRaAFLdA
26	AFK37127.1	------------------------sVADIVTpeFFNGIIDQADaSCAGKNFYSRDAFLNA
27	XP_0125709	TGCqQGPCFgGQNPPnsnt--NDVNVADIVTpqFFdsIIDQADSSCAGKNFYSRDAFLdA
28	XP_0202243	TGCqQGPCYnppSss------NnVNVADIVTpqFFNGIIDQADSgCAGKNFYSRDAFiNA
29	**KRH29572.1**	--------------------------dDIVTQDFFNsIIsQADdgCAGKNFYSRDAFLNA
30	KHN06178.1	--------------------------dDIVTQDFFNsIIsQADdgCAGKNFYSRDAFLNA
31	XP_0071319	hNSYprFGsLrneDDSKREvAAAFAHFTHEsGHFCYIEEIgGASrDYCDESNTEfPCvPs
32	BAT90863.1	hNSYhrFGnMnteDDSKREvAAAFAHFTHETGHFCYIEEIgGASrDYCDESNiEfPCAPN
33	XP_0226428	hNSYhrFGsLsSdDDSKREvAAAFAHFTHETGHFCYIEEIgGASrDYCDESNiEfPCAPN
34	XP_0256863	LNSYdQFGnLdtlDDSKREIAAAFAHFTHETGHFCYIEEIDGASrDYCDEtNTEYPCnPN
35	XP_0161870	LNSYdQFGnLdtlDDSKREIAAAFAHFTHETGHFCYIEEIDGASrDYCDEtNTEYPCnPN
36	XP_0159520	LNSYdQFGnLdtlDDSKREIAAAFAHFTHETGHFCYIEEIDGASrDYCDEtNTEYPCnPN
37	XP_0194143	LsSYNeFGRtGttDDSKREIAAAFAHFTHETGHFCYIEEIDGASKDYCeESNTqYPCAPN
38	PNY03482.1	LNSYNQFGRsGSvDDSKREIAAAFAHFTHETGHFCYIEEIDGpSKDYCDEnNTqYPCvaN
39	ACL36992.1	LNSYNQFGRsGSlDDSKREvAAAFAHFTHETGH-CYtEEIDGpSKDYCDEnNTEwPCAPN
40	XP_0035975	LNSYNQFGRsGSlDDSKREvAAAFAHFTHETGHFCYtEEIDGpSKDYCDEgNTEwPCAPN
41	AFK37127.1	LNSYNQFGsLdSlDDSKREIAAAFAHFTHETGHFCYIEEIDGASKDYCDEtNTEYPCAPN
42	XP_0125709	LNSYNQFGRVGlveDSKREIAAAFAHFTHETGHFCYIEEIDGASKDYCDESNTEYPCAtN
43	XP_0202243	LsSYdQFGRIGSeDDSKREIAAAFAHFTHETGHFCYIEEIDGASKDYCDEdNTqYPCvPN
44	**KRH29572.1**	hNSYNeFGRLGnqDDSKREvAAAFAHFTHETGHFCYIEEInGASgDYCDESNTEYPCAPN
45	KHN06178.1	hNSYNeFGRLGnqDDSKREvAAAFAHFTHETGHFCYIEEInGASgDYCDESNTEYPCAPN
46	XP_0071319	KGYYGRGPIQLSWNFNYGPAGqNIGFDGLNAPETVANDPVVSFKTALWYWMEfVRPVmNQ
47	BAT90863.1	KGYYGRGPIQLSWNFNYGPAGqNIGFDGLNAPqTVANDPVVSFKTALWYWMEfVRPVmNQ
48	XP_0226428	KGYYGRGPIQLSWNFNYGPAGqNIGFDGLNAPETVANDPVVSFKTALWYWMEfVRPVIhl
49	XP_0256863	KGYYGRGPIQiSWNFNYGPAGKNIGFDGLNAPETVANDPVVaFKTgLWYWMENVRPVVsQ
50	XP_0161870	KGYYGRGPIQiSWNFNYGPAGKNIGFDGLNAPETVANDPVVaFKTgLWYWMENVRPVVsQ
51	XP_0159520	KGYYGRGPIQiSWNFNYGPAGKNIGFDGLNAPETVANDPVVaFKTgLWYWMENVRPVVsQ
52	XP_0194143	KGYYGRGPIQLSWNFNYGPAGesnGFDGLNsPETVANDPVVSFKTALWYWMqhVhPVINQ
53	PNY03482.1	KGYYGRGPIQiSWNFNYGPAGKdIGFDGLNsPETVANDPtVSFKTALWYWMnNVhsVVsQ
54	ACL36992.1	KGYYGRGPIQLSWNyNYGPAGrdnGFDGLNsPETVANDaVVSFKTALW-WMnNVhgVINQ
55	XP_0035975	KGYYGRGPIQLSWNyNYGPAGrdnGFDGLNsPETVANDPtVSFKTALWYWMnNVhgVINQ
56	AFK37127.1	KGYYGRGPIQLSWNFNYGPAGKsndFDGLNAPETVANDPVVSFKTALWYWMqfVRPVlsQ
57	XP_0125709	KGYYGRGPIQLSWNyNYGaAGKdnGFDGLNsPETVANDPVVSFKTALWYWMkhVhPVINQ
58	XP_0202243	KGYYGRGPIQLSWNFNYGPAGqsnGFDGLNsPETVANDPVVSFKTALWYWMqhVRPVINQ
59	**KRH29572.1**	KaYYGRGPIQLSWNFNYGPAGqsIGFDGLNAPETVANDPVVSFKTALWYWMEhVRPVINQ
60	KHN06178.1	KaYYGRGPIQLSWNFNYGPAGqsIGFDGLNAPETVANDPVVSFKTALWYWMEhVRPVINQ
61	XP_0071319	GFGATIRAINGQLECDqgNPaTVQARVNhYThYCSQLGVAPGDNLyC-------------
62	BAT90863.1	GFGATIRAINGyLECnqgNPeTVnARVyYYTQYCSQLGVAPGDNLTC-------------
63	XP_0226428	GFGATIkAINGyLECnqgNPdTVnARVyYYTQYCSQLGVAPGDNLTCseisilseyglnc
64	XP_0256863	GFGATIRAINGQLECDGANPTTVQARVNYYkQYCSQLGVdPGDNLTC-------------
65	XP_0161870	GFGATIRAINGQLECDGANPTTVQARVNYYkQYCSQLGVdPGDNLTC-------------

66 XP_0159520 GFGATIRAINGQLECDGANPTTVQARVNYYkQYCSQLGVdPGDNLTC-------------
67 XP_0194143 GFGATIRAINGaLECDGgNPaTVQARVNYYTQYCSQLGVAtGDNLTC-------------
68 PNY03482.1 GFGATIRAINGkLECDGANPTTVQARVdYYkQYCSQLGVsPGDNLTC-------------
69 ACL36992.1 GFGATIRAINGrLECDGANPSTVQtRVgYYTQYCSeLGVAPGDNLTC-------------
70 XP_0035975 GFGATIRAINGrLECDGANPSTVQtRVgYYTQYCSeLGVAPGDNLTC-------------
71 AFK37127.1 GFGATIRAINGQLECDGANsnTVQARVNYYTQYCSQLGVAPGDNLTr-------------
72 XP_0125709 GFGATIRAINGQLECDGANPnTVQARVsYYTQYCSQLGVAPGDNLTC-------------
73 XP_0202243 GFGATIRAINGQLECDGANPTTVQARVNYYTeYCrQLGVAtGDNLTC-------------
74 **KRH29572.1** GFGATIRAINGrLECDGANPSTVQARVNYYTQYCSQLGVsPGDNLTC-------------
75 KHN06178.1 GFGATIRAINGrLECDGANPSTVQARVNYYTQYCSQLGVsPGDNLTC-------------

76 XP_0071319 --
77 BAT90863.1 --
78 XP_0226428 qscvfivitqfdrilhqamwnkkvqnmntgvtrvmvsflalmligcqqgacygtdsanhv
79 XP_0256863 --
80 XP_0161870 --
81 XP_0159520 --
82 XP_0194143 --
83 PNY03482.1 --
84 ACL36992.1 --
85 XP_0035975 --
86 AFK37127.1 --
87 XP_0125709 --
88 XP_0202243 --
89 **KRH29572.1** --
90 KHN06178.1 --

91 XP_0071319 --
92 BAT90863.1 --
93 XP_0226428 svadivtteffnnifdegdddadcpgknfysrqaflhalnsykqfgrsgsvddskreiaa
94 XP_0256863 --
95 XP_0161870 --
96 XP_0159520 --
97 XP_0194143 --
98 PNY03482.1 --
99 ACL36992.1 --
100 XP_0035975 --
101 AFK37127.1 --
102 XP_0125709 --
103 XP_0202243 --
104 **KRH29572.1** --
105 KHN06178.1 --

106 XP_0071319 --
107 BAT90863.1 --
108 XP_0226428 afahfthetqhfcyieesegeskdycdesksdeypcaadkeyygrgpiqlkwnynygaag
109 XP_0256863 --
110 XP_0161870 --
111 XP_0159520 --
112 XP_0194143 --
113 PNY03482.1 --
114 ACL36992.1 --
115 XP_0035975 --
116 AFK37127.1 --
117 XP_0125709 --
118 XP_0202243 --
119 **KRH29572.1** --
120 KHN06178.1 --

121 XP_0071319 --
122 BAT90863.1 --
123 XP_0226428 esigfdglkapetvgsdpvvsfktalwywtenvspvmeqgfgetiramkgevecdggnpd
124 XP_0256863 --
125 XP_0161870 --
126 XP_0159520 --
127 XP_0194143 --
128 PNY03482.1 --
129 ACL36992.1 --
130 XP_0035975 --
131 AFK37127.1 --

132	XP_0125709	--
133	XP_0202243	--
134	**KRH29572.1**	--
135	KHN06178.1	--

136	XP_0071319	--------------------------
137	BAT90863.1	--------------------------
138	XP_0226428	avqarvdyytqycsqlgvapgdnltc
139	XP_0256863	--------------------------
140	XP_0161870	--------------------------
141	XP_0159520	--------------------------
142	XP_0194143	--------------------------
143	PNY03482.1	--------------------------
144	ACL36992.1	--------------------------
145	XP_0035975	--------------------------
146	AFK37127.1	--------------------------
147	XP_0125709	--------------------------
148	XP_0202243	--------------------------
149	**KRH29572.1**	--------------------------
150	KHN06178.1	--------------------------
