# Supplementary material for: Identification and characterization of a new soybean promoter induced by Phakopsora pachyrhizi, the causal agent of Asian soybean rust
Source: BMC Biotechnol. 2021 Mar 25;21:27. doi: 10.1186/s12896-021-00684-9 (PMC7995590; doi:10.1186/s12896-021-00684-9)

a

Factor or Site Name	Location	strain	Signal Sequence	description
ARFAT	85	(+)	TGTCTC	AUX
AUXREPSIAA4	3278	(-)	KGTCCCAT	AUX
CAREOSREP1	19	(+)	CAACTC	GA
CAREOSREP1	2019	(-)	CAACTC	GA
GT1GMSCAM4	225	(-)	GAAAAA	pathogen salt induction
GT1GMSCAM4	566	(+)	GAAAAA	pathogen salt induction
GT1GMSCAM4	1644	(+)	GAAAAA	pathogen salt induction
GT1GMSCAM4	1922	(-)	GAAAAA	pathogen salt induction
GT1GMSCAM4	2331	(+)	GAAAAA	pathogen salt induction
GT1GMSCAM4	2533	(+)	GAAAAA	pathogen salt induction
GT1GMSCAM4	2769	(-)	GAAAAA	pathogen salt induction
GT1GMSCAM4	2860	(-)	GAAAAA	pathogen salt induction
GT1GMSCAM4	2878	(-)	GAAAAA	pathogen salt induction
GT1GMSCAM4	3031	(-)	GAAAAA	pathogen salt induction
MYBST1	122	(-)	GGATA	MYB TF (biotic/abiotic response)
MYBST1	798	(-)	GGATA	MYB TF (biotic/abiotic response)
MYBST1	1131	(-)	GGATA	MYB TF (biotic/abiotic response)
MYBST1	1819	(-)	GGATA	MYB TF (biotic/abiotic response)
MYBST1	1888	(-)	GGATA	MYB TF (biotic/abiotic response)
TATABOX2	332	(+)	TATAAAT	TATA box
WBBOXPCWRKY1	485	(+)	TTTGACY	W_box
WBBOXPCWRKY1	517	(+)	TTTGACY	W_box
WBBOXPCWRKY1	1276	(+)	TTTGACY	W_box
WBBOXPCWRKY1	3102	(-)	TTTGACY	W_box
WBBOXPCWRKY1	3233	(-)	TTTGACY	W_box
WBOXATNPR1	401	(-)	TTGAC	W_box
WBOXATNPR1	410	(+)	TTGAC	W_box
WBOXATNPR1	883	(-)	TTGAC	W_box
WBOXATNPR1	1672	(-)	TTGAC	W_box
WBOXHVISO1	117	(+)	TGACT	W_box
WBOXHVISO1	479	(-)	TGACT	W_box
WBOXHVISO1	504	(+)	TGACT	W_box
WBOXHVISO1	696	(+)	TGACT	W_box
WBOXHVISO1	1354	(+)	TGACT	W_box
WBOXHVISO1	3169	(-)	TGACT	W_box
WBOXNTERF3	1380	(-)	TGACY	W_box
WRKY71OS	480	(-)	TGAC	W_box
WRKY71OS	1175	(-)	TGAC	W_box
WRKY71OS	1589	(-)	TGAC	W_box
WRKY71OS	1802	(-)	TGAC	W_box
WRKY71OS	2385	(+)	TGAC	W_box

b

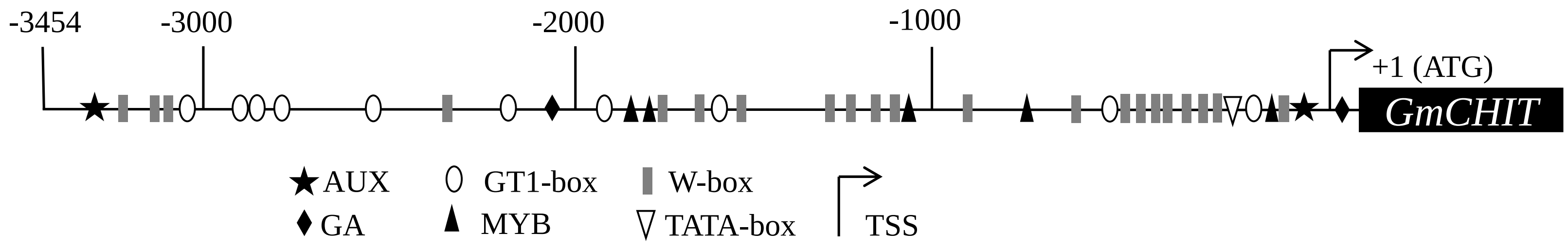

Supplement: Supplementary file 7 — Additional file 7: Figure S6. Detection of GFP fluorescence in stable transgenic soybeans. Leaves of two T1 lines (129 and 133) transformed with the pGmCHIT1:GFP construction were observed using a dissection scope (Leica Z16 APO) under GFP filter and bright light at 24 and 72 hours after. P. pachyrhizi inoculation (+) or mock treatment (-). Arrows indicate the inoculation spots. Bar-scales represent 200 μm. [file 12896_2021_684_MOESM7_ESM.pdf]
